# Sustain-Release of Various Drugs from Leucaena Leucocephala Polysaccharide

**DOI:** 10.4103/0975-1483.62207

**Published:** 2010

**Authors:** S Jeevanandham, M Sekar, D Dhachinamoorthi, M Muthukumaran, N Sriram, J Joysaruby

**Affiliations:** *Department of Pharmaceutics, Santhiram College of Pharmacy, Andhra Pradesh, India*; 1 *Department of Pharmaceutics, QIS College of Pharmacy, Andhra Pradesh, India*

**Keywords:** Kernel powder, Leucaena leucocephala, mucoadhesives

## Abstract

This study examines the sustained release behavior of both water-soluble (acetaminophen, caffeine, theophylline and salicylic acid) and water-insoluble (indomethacin) drugs from Leucaena leucocephala seed Gum isolated from Leucaena leucocephala kernel powder. It further investigates the effect of incorporation of diluents like microcrystalline cellulose and lactose on release of caffeine and partial cross-linking of the gum (polysaccharide) on release of acetaminophen. Applying exponential equation, the mechanism of release of soluble drugs was found to be anomalous. The insoluble drug showed near case II or zero-order release mechanism. The rate of release was in the decreasing order of caffeine, acetaminophen, theophylline, salicylic acid and indomethacin. An increase in release kinetics of drug was observed on blending with diluents. However, the rate of release varied with type and amount of blend in the matrix. The mechanism of release due to effect of diluents was found to be anomalous. The rate of release of drug decreased on partial cross-linking and the mechanism of release was found to be super case II.

## INTRODUCTION

Hydrophilic matrices are an interesting option when developing an oral sustained-release formulation. They can be used for controlled release of both water-soluble and water-insoluble drugs. The release behavior of drugs varies with the nature of the matrix and it is the complex interaction of swelling, diffusion and erosion process.[1] Release of drugs from such matrices can be controlled through their physical properties, the correct choice of gelling agent and setting up the conditions for fabrication.[2] Among hydrophilic polymers, polysaccharides are the choice material due to their nontoxicity and acceptance by regulating authorities.[3] Polysaccharides like cellulose ethers,[4] xanthan gum,[5] scleroglucan,[6] locust bean gum[7] and gaur gum[8] are some of the natural polysaccharides which have been evaluated in the hydrophilic matrix for drug delivery system. Although Leucaena leucocephala seed polysaccharide (LLSP) is used as ingredient in food material, in pharmaceuticals has not been evaluated as hydrophilic drug delivery system. LLSP is a galactoxyloglucan isolated from seed kernel of Leucaena leucocephala. It possesses properties such as high viscosity, broad pH tolerance, and adhesivity.[9] This led to its application as stabilizer, thickener, gelling agent and binder in food and pharmaceutical industries. In addition to these, other important properties of LLSP have been identified recently. They include non-carcinogenicity,[10] mucoadhesivity, biocompatibility,[11] high drug holding capacity,[12] and high thermal stability.[13] This led to its application as excipient in hydrophilic drug delivery system.[11–12] Since LLSP is an important excipient, the present study was undertaken to elucidate release kinetics of both water-soluble and water-insoluble drugs from this matrix. In order to predict and correlate the release behavior of drugs from the hydrophilic matrix, it is necessary to fit into a suitable model. The commonly adopted model for understanding such behavior from hydrophilic matrices is simple exponential equation.[14] This model facilitates the understanding of mode of release like: Whether the release is due to only diffusion or only erosion, or due to both diffusion and erosion. This model has been used for this study.

## MATERIALS AND METHODS

### Materials

Leucaena leucocephala seeds have been collected from the area of Kurnool district Andhra Pradesh (India); acetaminophen and caffeine were obtained as gift sample from Tablets India Limited, Chennai. Salicylic acid from Qualigens (India), indomethacin, and theophylline anhydrous from Sigma Chemicals Company were purchased. Microcrystalline cellulose, lactose monohydrate, and magnesium stearate were purchased from Central Drug House (India). Absolute ethanol, diethyl ether, petroleum ether, glacial acetic acid, epichlorohydrin and acetone from Qualigens (India) and sodium hydroxide from E-Merck (India). All the chemicals used were of analytical grade.

### Isolation of leucaena leucocephala seed polysaccharide

LLSP was prepared following methods by Rao *et al*.,[15] in three batches on a laboratory scale. To 20 g of Leucaena leucocephala kernel powder, 200 ml of cold distilled water was added and slurry was prepared. The slurry was poured into 800 ml of boiling distilled water. The solution was boiled for 20 min under stirring condition in a water bath. The resulting thin clear solution was kept overnight so that most of the proteins and fibers settled out. The solution was then centrifuged at 5000 rpm for 20 min. The supernatant was separated and poured into twice the volume of absolute ethanol by continuous stirring. The product was pressed between felt. The precipitate was washed with absolute ethanol, diethyl ether, and petroleum ether and then dried at 50-60°C under vacuum. The dried material was ground and sieved to obtain granules of different particle size range. The particle size range of 150-75 μm was used for preparation of tablets.

### Characterization of leucaena leucocephala seed polysaccharide by C13 NMR and X-ray diffraction

#### N.M.R. Spectroscopy

The ^13^C N.M.R spectrum was recorded for LLSP solution in D_2_O. The sample was dissolved by heating.

#### X-ray diffraction

Diffraction pattern of the powdered LLSP sample was recorded with an X-ray diffractometer (CECRI, Tuticorin). X-ray diffraction was performed at room temperature (30°C) with a diffractometer; target, Cu (λ = 1.54 Å), filter, Ni; Voltage, 40 kV; current 30 mA; time constant 10 mm/s; scanning rate 2°/min; measured from 10-35° at full scale 200.

### Cross-linking of leucaena leucocephala seed polysaccharide

LLSP was partially cross-linked with epichlorohydrin.[16] LLSP 10 g (soaked in water) and sodium hydroxide (50 ml, 1 N, 54°C) were mixed with a glass rod. After homogenization (15 min), 0.5 ml epichlorohydrin (6 g/100 g of LLSP) was slowly added with continuous homogenization (15 min). The gel was then neutralized with acetic acid and washed three times through a sintered glass filter with a solution of water/acetone (60:40 v/v). In the final step, the resulting solid gel was washed with pure acetone over a filter. The polymer was air dried at room temperature for 72 h and stored in airtight container. After granulation, granular fractions between 75 and 250 mm were used for preparation of tablets. A cross-linked polysaccharide was prepared in three batches.

### Preparation of tablet

The total weight of the tablets (without magnesium stearate) were 250 mg for drug: Polymer ratio of 1:4 and 300 mg fordrug: Polymer ratio of 1:2. The ingredients [Table 1] were mixed in mixer for 5 min before and 5 min after addition of magnesium stearate (lubricant). The tablets were prepared using single-punch hand operated tablet machine (Cadmach) fitted with flat-faced punches at 5 tons compression pressure for 30 seconds. The diameter of the tablet was 13 mm and was kept constant throughout the experiment.

**Table 1:** Formulations of various Leucaena leucocephala seed polysaccharide matrices

Ingredients	Drug type (mg/tablet)	Cross linker (mg/tablet)	Diluents (mg/tablet)
Drug Substance^a^	50	50/100	50
LLSP^b^	200	0	180/160/140/120/100/80
Cross-linked LLSP	0	200	0
Lactose/MC^c^	0	0	20/40/60/80/100/120
Magnesium Stearate	2.5	2.5/3	2.5

a Caffeine/acetaminophen/theophylline/salicylic acid/indomethacin

b Leucaena Leucocephala seed polysaccharide

c Microcrystalline cellulose

### Equilibrium swelling study

Equilibrium swelling volume[16] of partially cross-linked LLSP powder and tablet was measured in water at 37°C. The drug-free tablets of 250 mg each (or 250 mg of powder) were placed in a 25 ml graduated cylinder to which 10 ml of water was added. After 48 h, the equilibrium swelling volume was read directly as the volume of the gel bed. The swelling was expressed as swollen volume per unit weight of initial dry material (ml/g).

### *In vitro* drug release study

Single face release experiments were performed at 37°C. The sample holder was immersed in 900 ml distilled water for caffeine, acetaminophen, theophylline, salicylic acid, and phosphate buffer pH 7.2 for indomethacin. Sink condition was followed for the whole experiment, as the volume of dissolution medium was more than 10 times the solubility of drugs in the dissolution medium. Agitation of 100 rpm was provided and concentration of drug in the dissolution medium was measured as a function of time. The concentrations of caffeine, acetaminophen, theophylline, salicylic acid, and indomethacin were determined by monitoring the UV absorbance of the dissolution medium at 273, 242, 271, 297 and 318 nm, respectively [Table 2]. The experiments were done for each batch and average values are reported.

**Table 2:** List of model drugs used for preparation of matrix tablet

Drug type	Solubility in water at 37°C (mg/ml)	Detection wavelength (nm)
Caffeine anhydrous	37.0	273
Acetaminophen	18.9	242
Theophylline anhydrous	9.9	271
Salicylic acid	3.1	297
Indomethacin	0.9	318

Prepared in phosphate buffer - pH -7

### Model used for analysis of drug-release kinetics

The dissolution data were fitted according to the well. known exponential equation,[14] which is often used to describe the drug-release behavior from polymeric systems.

(1) $$M_{t}/M_{\infty }\;=\;{\mathrm{kt}}^{n}$$

where M_*t*_/M_∞_ is the fractional release of the drug, ‘*t*’ is the release time, ‘*k*’ is a constant incorporating structural and geometric characteristic of the release device (tablets) and *n* is the release exponent indicative of the mechanism of release. Table 3, shows an analysis of diffusional release mechanism obtained by varying the *n* values.[17] The *n* values used for analysis of the drug release mechanism from the tablets were determined from log (M_*t*_/M_∞_) vs. log (*t*) plots.

**Table 3:** Variation of *n* values with the mechanism of diffusion

*n*	Mechanism	dM_*t*_/dt dependence
0.5	Fickian diffusion	*t*^−0.5^
0.5 < *n* > 1.0	Anomalous diffusion	*t*^*n*–1^
1.0	Case II transport	Zero order
*n* > 1.0	Super case II transport	*t*^*n*–1^

## RESULTS AND DISCUSSION

### Characterization of leucaena leucocephala seed polys accharide

**Figure 1:**
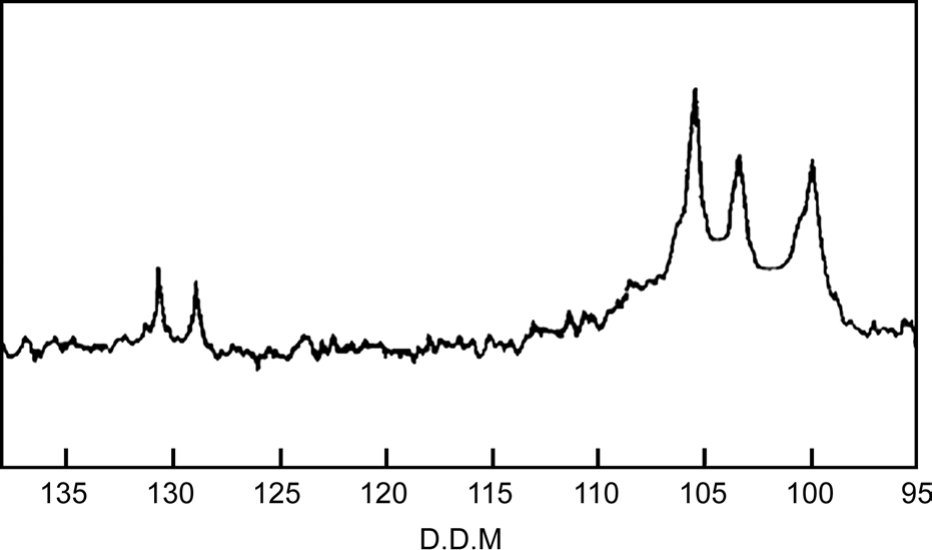
^13^C-N.M.R. spectrum of leucaena leucocephala seed polysaccharide

^13^C N.M.R: The^13^C N.M.R spectrum of LLSP is shown in Figure 1. The spectrum shows C-1 signals at 105.4, 103.4, and 100.0 ppm that are assigned to galactose, glucose, and xylose residues, respectively. The result complies with the reported values.[18]

**Figure 2:**
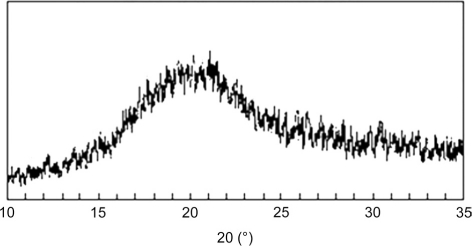
X-ray diffraction pattern of leucaena leucocephala seed polysaccharide

X-ray diffraction analysis: The X-ray diffraction pattern [Figure 2] of LLSP did not show any characteristic peak, which indicates that the structure is completely amorphous. The result confers with the X-ray diffraction study of Leucaena leucocephala xyloglucan.[19] The results show that the isolated polysaccharide has similar behavior to that reported by others. Thus polysaccharide isolated can be used in the following study.

### Effect of solubility of drug

**Figure 3:**
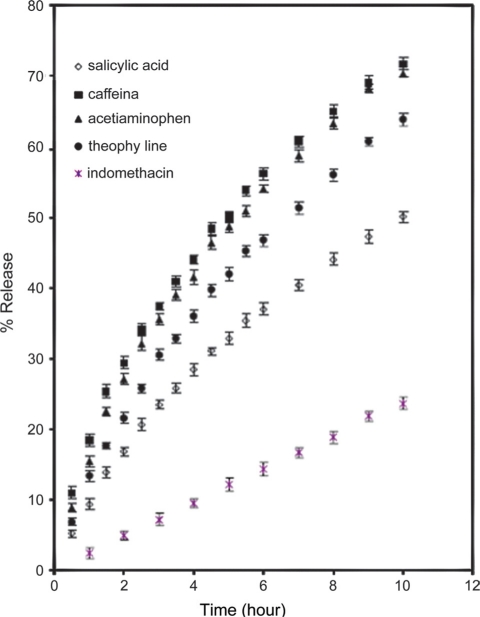
Release profile of drugs of different solubility from leucaena leucocephala seed polysaccharide tablets (mean ± SD; n = 3)

Figure 3 shows the release of drugs from LLSP matrices.

The release of drug depends not only on the nature of the matrix, but it also depends upon the solubility of the drug. So the release of drugs with different solubility parameters such as caffeine, acetaminophen, theophylline, salicylic acid, and indomethacin was studied [Table 2]. The intrinsic dissolution of the drugs in the dissolution medium was determined. The procedure followed was that reported by Tarara *et al*.[20] The 1 g of drugs in 10 ml of dissolution medium were kept on a shaker at 37°C for 42 h. A 5 ml of solutions were centrifuged at 5000 rpm for 15 min. Then passed the supernatant through Millipore filter. The absorbances were measured at respective absorbance value and solubility values were calculated [Table 2]. The rates of release of drugs from the matrices [Figure 3] are in decreasing order of the solubility parameters. The mechanism of release of soluble drugs is anomalous (*n* > 0.5), while indomethacin (water insoluble drug) showed behavior of near case II or zero-order release [Table 4]. This indicates that the release is controlled by both diffusion and erosion phenomena. The latter dominates the release as the solubility of drug in water decreases and vice versa.[20] About 50% of total loading of drug releases in 5, 5.5, 7 and 10 h for caffeine, acetaminophen, theophylline, and salicylic acid, respectively. The total release percent of soluble drugs in 5 h decreases from 50% to 32% as the solubility of drug in water decreases. The total release of indomethacin in the first 5 h is about 10% of total load of the tablet. The rate of release of drugs decreases with decrease in solubility of the drugs. It is because the water dissolves the drug at the surface first, and then penetrates the matrix via pores, bringing about a gelling of the polymer. Dissolved drug is then released by diffusion through the gel and finally the release rate falls as the water reaches the center due to decreased drug concentration to less than its solubility.[1,21] The solubility of indomethacin in aqueous medium (phosphate buffer) is very low. Due to the slow erosion of the matrix and low solubility, the amount of drug released is also less. The value of n varies from anomalous to near zero order as the solubility of drugs decreases [Table 4].

**Table 4:** The *n* value of the formulations containing drug type with D:P ratio of 1:4 and cross linker with D:P ratio of 1:2 and 1:4

Formulation	*n*
Drug type	
Caffeine	0.60
Acetaminophen	0.66
Theophylline	0.71
Salicylic acid	0.73
Indomethacin	0.98
Cross-linker	
1:2	1.24
1:4	1.25

### Effect of diluents

**Figure 4:**
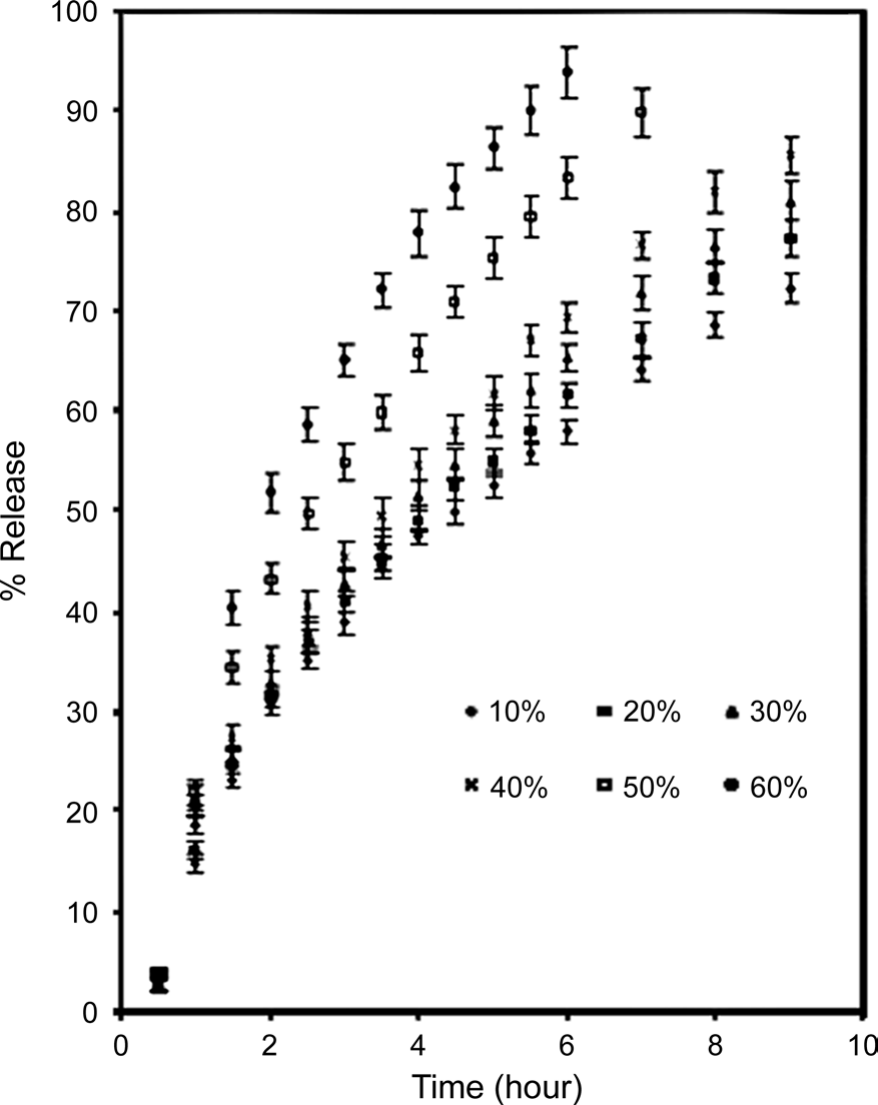
Effect of replacing leucaena leucocephala seed polysaccharide with lactose on release of caffeine (mean ± SD; n = 3)

**Figure 5:**
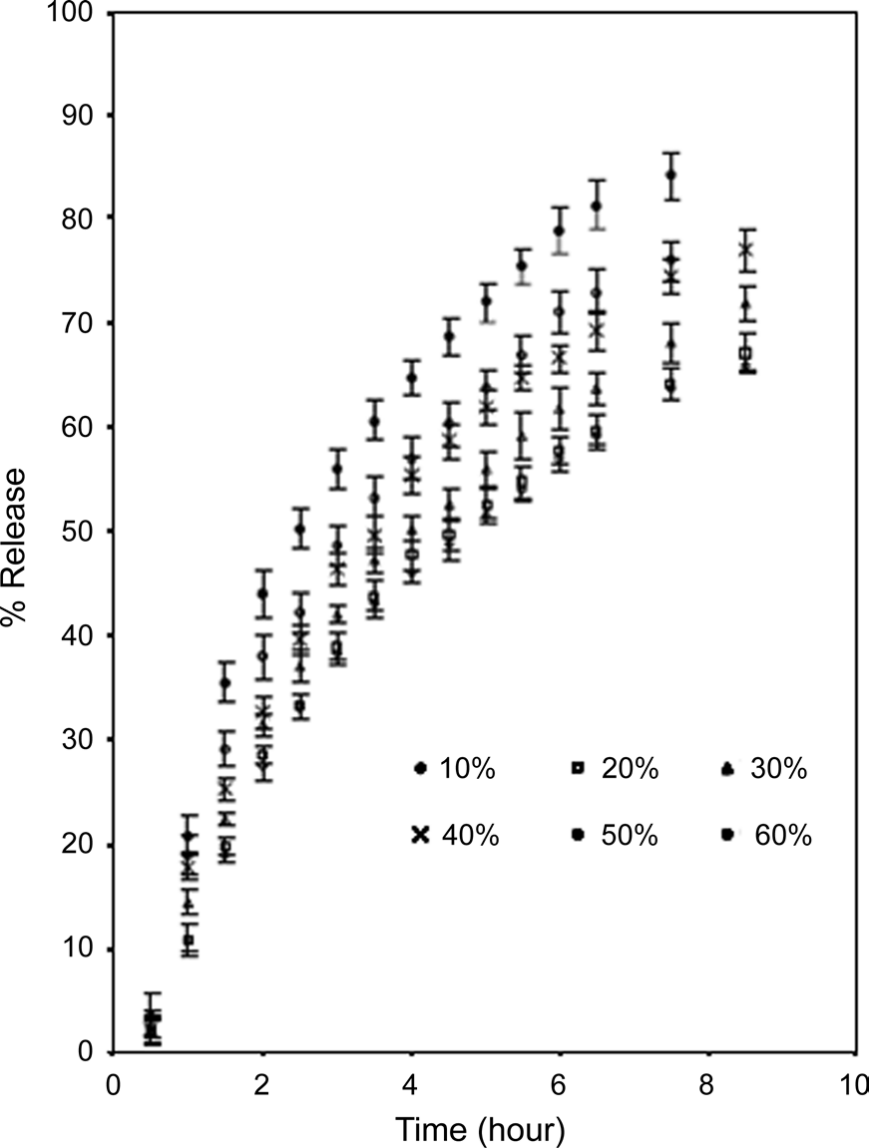
Effect of replacing leucaena leucocephala seed polysaccharide with microcrystalline cellulose on release of caffeine (mean ± SD; n = 3)

Figures 4 and 5 show the effect of diluents. Two materials were chosen for this purpose, namely lactose and microcrystalline cellulose. The former is watersoluble, while the latter is relatively hydrophobic. These two separately were blended with LLSP and caffeine [Table 1].

The mechanisms of release of caffeine from the blends were found to be anomalous [Table 5].

**Table 5:** The n value of formulations containing D:P ratio of 1:4 when replacing the polymer with different amount of lactose and microcrystalline cellulose

Replacement	*n*	t_50_
Lactose (%)		
0	0.60	50.0
10	0.59	4.5
20	0.60	4.0
30	0.61	3.75
40	0.61	3.5
50	0.61	2.5
60	0.59	2.0
MC (%)		
0	0.60	5.0
10	0.59	4.5
20	0.56	4.5
30	0.56	4.0
40	0.58	3.5
50	0.57	3.15
60	0.52	2.5

As the percentage of diluents increased, the kinetics of release also increased. This may be due to structural reorganization of the hydrophilic polysaccharide matrix.[3,22,23] The lactose being water soluble would undergo dissolution and that may result in reduction in the tortuosity and or gel strength of the polymer. The T_50_ value of lactose and microcrystalline cellulose were nearly same up to 40%, and above 40% the rate of release was faster in case of lactose [Table 5]. The slow release could be due to reported interaction of Leucaena leucocephala seed polysaccharide with microcrystalline cellulose.[19]

### Effect of partial cross-linking of matrix

**Figure 6:**
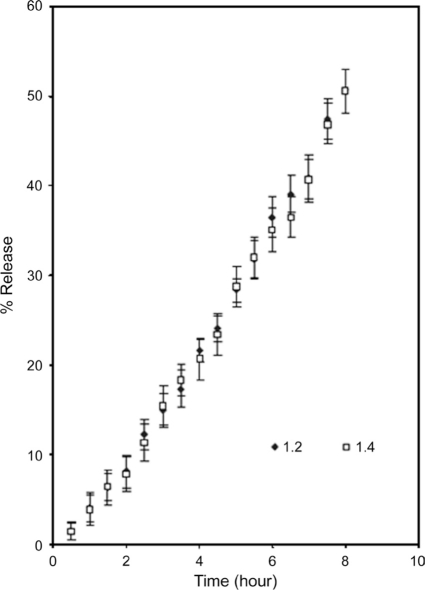
Release profile of acetaminophen from cross-linked leucaena leucocephala seed polysaccharide tablets (mean ± SD; n = 3)

The partially cross-linked LLSP powder and tablet had equilibrium swelling volume of 22 ml/g and 12 ml/g, respectively. This shows that intergranular hydrogen bonds exists in the tablets due to compression like that of crosslinked amylase.[16] The mechanism of drug (acetaminophen) release from the two formulations of cross-linked LLSP was found to be super case II [Table 3], and dissolution T_50_ value for drug was 8 h [Figure 6]. The release could be sustained at a constant rate for longer period than with uncross-linked material. The effect of drug loading had no effect on the percent of release [Figure 6]. The slow rate of drug release could be due to slow water penetration due to the presence of numerous intergranular hydrogen bonds and presence of gel barrier.[16] This shows that by controlling degree of cross-linking, the release kinetics can be optimized to desire design.

## CONCLUSION

Leucaena leucocephala seed polysaccharide can be used for controlled release of both water-soluble and water-insoluble types of drugs. Zero-order release can be achieved taking sparingly soluble drug like indomethacin from LLSP. The rate of release can be controlled by using suitable diluents like lactose and microcrystalline cellulose. For water-soluble drugs, the release amount can also be controlled by partially cross linking the matrix. The extent of release can be varied by controlling degree of cross-linking.
